# Transforming neurophysiological data into measurable stress metric using machine learning-based stress quantification framework for smart factory workers

**DOI:** 10.3389/fnrgo.2026.1871817

**Published:** 2026-10-21

**Authors:** Arshia Arif, Zohreh Zakeri, Ahmet Omurtag, Philip Breedon, Azfar Khalid

**Affiliations:** Department of Engineering, School of Science & Technology, Nottingham Trent University, Nottingham, United Kingdom

**Keywords:** EEG, fNIRS, human robot collaboration (HRC), machine learning, mental stress quantification, occupational health and safety

## Abstract

**Introduction:**

Mental stress is a common issue in demanding occupational setups, such as smart industrial settings, particularly when working with robots, being one of the primary reasons for decreased performance and productivity. Estimating and evaluating stress are critical for worker safety, performance, and overall well-being. A novel integrated framework is proposed in this research for continuous cognitive stress-level estimation that combines neuroimaging (EEG and fNIRS), gaze tracking and machine learning.

**Methodology:**

A factory workers' stress assessment experiment is designed and implemented, which employs physiological, behavioral and subjective measures to assess mental stress from different perspectives. Physiological features extracted from multimodal neuroimaging data are used for training supervised classification and regression models, while gaze, behavioral, and subjective measures provide complementary evaluation of worker stress. To further optimize the pipeline, multiple feature selection algorithms are tested, followed by ensemble learning approaches, and the best model is chosen for continuous stress-level estimation.

**Results and discussion:**

After implementing the novel stress quantification framework for the factory workers' stress assessment experiment, the ensemble learning approach produced the best results for both regression (RMSE: 10.86) and classification (accuracy: 84.1%) techniques using the STAI score as the target. The behavioral and subjective measures demonstrate the effect of varying process variables (light, noise, task speed, and complexity) during the experiment. Multimodal data, machine learning, and other computational approaches are integrated in this study to estimate continuous cognitive stress levels, utilizing the proposed framework presented in this research, thereby bridging the gap between research and practical application. The proposed multi-domain framework provides a promising solution for monitoring worker well-being in occupational settings.

## 1 Introduction

Digitization and automation have reshaped manufacturing systems, leading to smart factories where Human-Robot Collaboration (HRC) is critical for optimal and desired productivity, flexibility, and quality standards (Othman and Yang, 2023). In these settings, although combining human cognitive strengths with robotic precision offers significant benefits (Ajoudani et al., 2018), constant interaction with smart machines and adapting to dynamic robot behavior can also create psychological challenges (De Simone et al., 2022). These factors may increase cognitive stress among workers in human-robot collaborative environments (De Simone et al., 2022; Sun et al., 2023).

Technostress, mental strain caused by rapid technological change (Rohwer et al., 2022), is particularly evident in human-robot collaborative environments. Although cobots are designed for safety and efficiency (Vagaš et al., 2020; Patil et al., 2023), their close proximity can pose psychological pressure (Sun et al., 2023). Continuous monitoring of robot behavior, fear of unexpected malfunctions, and the need to synchronize with automated systems can induce anxiety. Trusting complicated automated systems for personal safety and productivity can cause mental overload, fatigue, and heightened stress, affecting workers' cognitive health and job satisfaction (Liu et al., 2021).

In smart factories, incorporating neuroergonomics and human factors is crucial to create human-centered, stress-free workspaces for employees collaborating with cobots (Blandino, 2023; Zakeri et al., 2021). However, the rapid evolution of smart manufacturing has raised concerns about worker health, safety, and prevalent ergonomic problems (Hijry et al., 2024). Improving employee wellbeing remains an ongoing challenge for both SMEs and large industrial organizations (Gualtieri et al., 2020).

Conventionally, mental stress has been evaluated using subjective measures, such as self-report questionnaires and rating scales (Cohen et al., 1983; Braarud, 2020; Legler et al., 2023; Matthews et al., 2015), as well as behavioral measures, including task accuracy, reaction time, speech features, facial expressions, and gaze behavior (Aylward and Robinson, 2017; Braarud et al., 2021; Jaiswal, 2023; Upasani et al., 2024). Behavioral indicators provide insight into observable stress-related responses and have been widely used to assess cognitive performance, attention, and visual behavior under stress (Aylward and Robinson, 2017; Braarud et al., 2021; Jaiswal, 2023; Upasani et al., 2024; Khode et al., 2022; Huang et al., 2016). Subjective measures, including the Perceived Stress Scale (PSS) (Cohen et al., 1983), NASA Task Load Index (NASA-TLX) (Braarud, 2020), Instantaneous Self-Assessment (ISA) (Leggatt, 2023), State-Trait Anxiety Inventory (STAI) (Legler et al., 2023), Technology Acceptance Model (TAM) (Rossato et al., 2021), and Positive and Negative Affect Schedule (PANAS) (Can et al., 2019; Tran, 2020), have been extensively used to evaluate perceived stress, cognitive workload, emotional state, and human-robot interaction experiences across diverse occupational and collaborative environments.

While conventional measures, including subjective and behavioral measures, provide useful insights, physiological measures offer a more objective assessment by recording stress-induced bodily changes (Setz et al., 2009; Shu et al., 2018). Common physiological indicators include heart rate variability (HRV) (González Ramírez et al., 2023; Tiwari et al., 2021), cortisol levels (Jerčić et al., 2020), electrodermal activity (EDA), body temperature, and ocular metrics such as pupil dilation and blink rate (Gomes et al., 2023; Jerčić et al., 2020; Kumar et al., 2021). Advances in sensing technology and data analytics have facilitated the integration of physiological signals, such as electrocardiography (ECG) (Zhang et al., 2021), facial expressions, and speech features (Zhang et al., 2022), into multimodal stress-evaluation frameworks, often combined with machine learning for continuous, real-time monitoring (Ayres et al., 2021; Vanneste et al., 2021). Among these indicators, brain signals, including electroencephalography (EEG) (Katmah et al., 2021; Zakeri et al., 2023) functional magnetic resonance imaging (fMRI) (Causse et al., 2022) and functional near-infrared spectroscopy (fNIRS) (Molina-Rodríguez et al., 2023; Zakeri et al., 2023) are important for understanding the neurological mechanisms underlying mental stress.

The research gap uncovered from the existing literature is that many researchers are attempting to detect, analyze, and comprehend mental stress using various methods; however, to the best of the authors' knowledge, few studies have investigated continuous cognitive stress-level estimation, especially in industrial human-robot collaborative environments. While extensive research has been carried out to examine the causes and consequences of cognitive stress in various occupational contexts, most of the existing work has focused on qualitative assessments, self-reported surveys, and observational studies. Recent improvements have also enabled the detection or classification of stress levels (e.g., low, medium, high) based on physiological and behavioral measures, which has exciting implications in a range of professions. However, the majority of this research attempts to identify stress presence or classify stress states in a binary or categorical manner, rather than providing a continuous, real-time tracking of stress intensity as a quantifiable and measurable factor. Although such approaches are useful for detecting broad stress states, they provide limited information regarding gradual variations in worker stress. Hence, the primary objective of this research is to develop and implement a novel, comprehensive framework for evaluating and quantifying cognitive stress to benefit both employers and employees. In this study, an experiment is designed to quantify factory workers' cognitive stress using EEG and fNIRS neuroimaging (Arif et al., 2024), along with gaze data such as pupil diameter. Error rate is used as the behavioral indicator, while the NASA Task Load Index (NASA-TLX) (Braarud, 2020) and State-Trait Anxiety Inventory (STAI) (Legler et al., 2023) scores provide subjective stress assessments (Ramadhana et al., 2021; Fauquet-Alekhine et al., 2016). NASA-TLX assesses perceived workload using characteristics such as mental demand, effort, and frustration (Braarud, 2020), whereas STAI assesses anxiety-related reactions that are strongly linked to perceived stress (Legler et al., 2023). Although increasing workload can contribute to increased stress levels (Ramadhana et al., 2021), workload and stress are separate variables. Additional gaze metrics, including fixation duration, number of visits, and visit duration, reflect behavioral responses.

Recent research has discovered that multimodal neuroimaging techniques can effectively assess workplace stress. Masri et al. (2026) for example, presented a workplace stress management approach that employed multimodal neuroimaging data fusion and binaural beat stimulation, demonstrating the efficacy of combining EEG and fNIRS measures for stress monitoring. Furthermore, Masri et al. (2024) conducted a thorough review of workplace stress assessment approaches and found neurophysiological sensing and machine learning to be promising approaches for stress evaluation in occupational contexts. These studies show a growing interest in multimodal neuroimaging-based stress assessment; however, few studies have explored continuous stress-level estimation in factory-inspired human-robot collaboration environments using multimodal physiological measurements combined with behavioral, gaze-based, and subjective assessments.

In industrial setups, stress levels can fluctuate constantly due to changing task demands, workload, and human-robot interactions. Thus, continuous stress prediction provides a more accurate and thorough depiction of workers' mental state by tracking incremental changes in stress intensity rather than allocating workers to fixed categories, allowing for more effective interventions. For example, a progressive rise in predicted stress levels may signal the need for workload redistribution, task adjustment, or brief recovery breaks before critical stress thresholds are reached. Furthermore, adaptive manufacturing systems and cobots may use continuous stress assessments to automatically adjust task pace, interaction frequency, or support levels based on the worker's stress condition. Therefore, compared to the discrete stress classes, continuous stress prediction can offer a more informative foundation for workplace decision-making and safety management.

Based on the literature review and identified research gap, the following hypotheses are formulated:

**H1:** Changes in the process variables and environmental conditions result in variations in workers' cognitive stress levels.
**H2:** A multimodal machine-learning-based framework incorporating brain signals can effectively estimate workers' cognitive stress levels.

This study aims to estimate factory workers' continuous cognitive stress level within a human-machine collaborative environment by simulating a production line and using multidimensional data, including behavioral, physiological, and subjective parameters. The novel aspect of this research lies in the development of a multimodal framework for continuous stress-level estimation in a human-robot collaboration environment using EEG and fNIRS measurements supported by complementary behavioral, gaze-based, and subjective assessments, moving beyond conventional binary and multiclass stress classification. The framework is trained using validated subjective stress assessments but allows future stress score estimation using physiological parameters alone, eliminating the requirement for subjective questionnaire administration during deployment. Another novelty of this study is the integration of a feature selection and ensemble learning approach within the proposed stress-estimation framework. Multiple machine-learning algorithms are evaluated to identify the best model, with improved accuracy (for the classification approach) and lower RMSE (for the regression approach), for continuous stress-level estimation in a human-robot collaborative environment. This approach has also reduced the bias for both classification and regression approaches. The interpretations from this research can be applied to any workspace where humans and cobots coexist.

## 2 Methodology

To facilitate the development of intelligent AI-based systems that detect and monitor mental stress, digitizing mental stress involves thoroughly quantifying individuals' mental states into a measurable format. In this study, stress quantification refers to the estimation of continuous stress levels using validated subjective stress ratings. These scores are administered as target variables for training supervised learning models based on physiological features derived from EEG and fNIRS signals. Therefore, the proposed approach focuses on estimating perceived stress severity rather than performing binary or multi-class stress classification.

Figure 1 depicts the eight-step pipeline used in this study to quantify cognitive stress. It starts with data collection and ends with a predictive model for cognitive stress. Participants‘ subjective self-reported stress levels, behavioral indicators, and objective physiological markers are initially used to collect data. This data is collected while participants perform stress-inducing cognitive activities, i.e., different versions of the Stroop test, ANT, N-back task or problem-solving IQ-style questions, that are designed to mimic real-world mental workload situations. The step after the data collection is the preprocessing phase, involving reduction in noise, artifacts, and discontinuities in raw physiological signals. Different physiological metrics typically require different preprocessing strategies. To obtain clean signals, preprocessing methods, depending on the nature of the physiological data, must be used. In the subsequent step, feature extraction, multidimensional features like statistical, frequency domain, and time domain features are extracted using the preprocessed data. The next phase, mapping subjective stress scores to corresponding physiological features, is the framework's highlight and novelty, as it is the primary notion being employed in this study to quantify cognitive stress. This mapping enables the generation of labeled data for use in supervised machine learning algorithms. This mapping establishes a significant link between self-reported ratings for mental stress and physiological data patterns. Following that, feature selection methods are administered to minimize dimensionality while increasing model accuracy and efficiency by determining the most relevant stress indicators. The chosen features are then used to train machine learning models capable of recognizing patterns linked to various stress levels. Several techniques are tested at this point to determine the optimal machine learning model for mental stress prediction. The stress predictions' robustness and bias are then improved using ensemble learning techniques. The combined results of different base learners are utilized to improve prediction accuracy while lowering the risk of overfitting.

**Figure 1:**
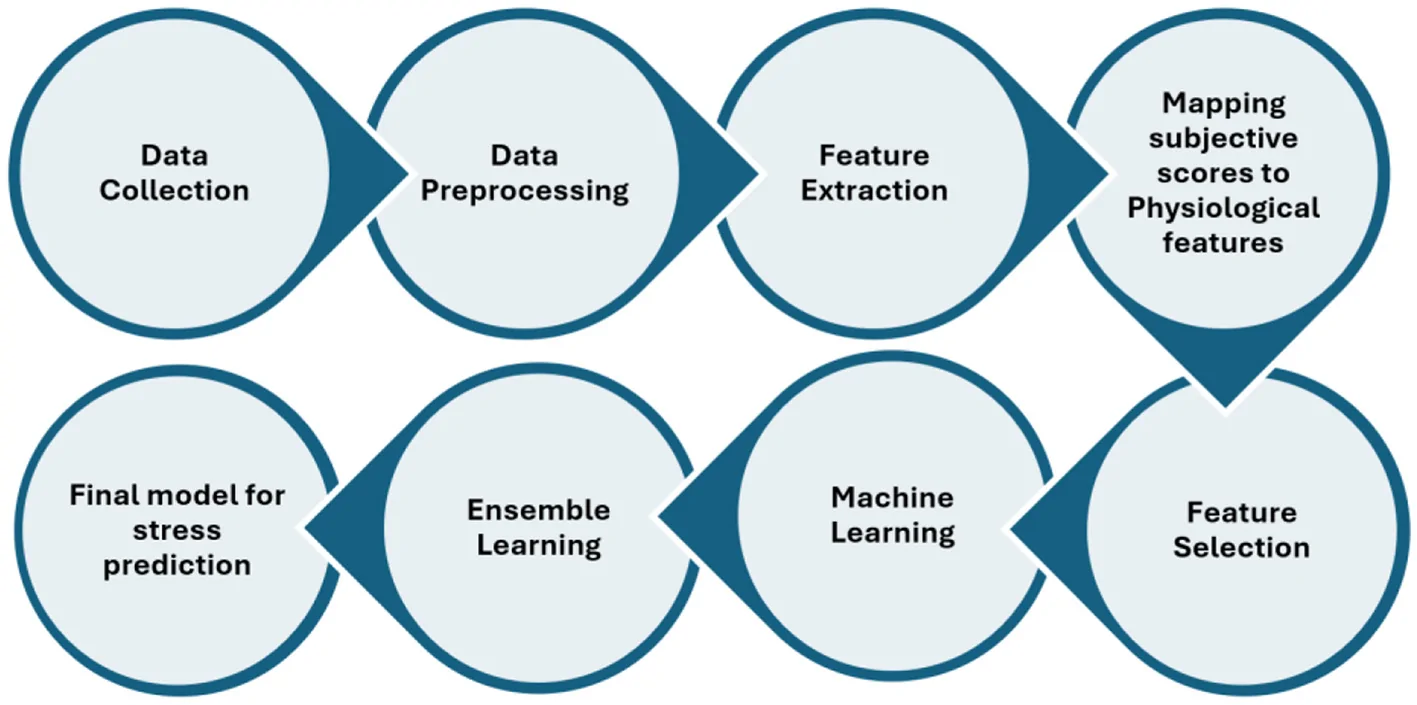
Flowchart of the proposed mental stress quantification framework. The main processing stages for the proposed stress quantification framework are illustrated.

The proposed multimodal stress assessment framework includes both predictive and support modalities. EEG and fNIRS signals are the primary inputs to machine-learning models, whereas subjective assessments (NASA-TLX and STAI scores) serve as labels for quantifying mental stress. In contrast, gaze-tracking metrics and behavioral performance measurements are used as complementary assessment modalities to characterize participants‘ responses across different stress settings and provide conclusive results alongside physiological findings. Although gaze and behavioral markers are not used as direct inputs to the predictive models, they contribute to the holistic multimodal evaluation by providing supporting information regarding participants' responses alongside the presence of stress-related variations in neurophysiological signals.

The ensemble model is ultimately tested and chosen as the framework's output, providing a predictive model that quantifies mental stress using multidimensional data. This model supports practical applications in real-time occupational stress monitoring. Overall, the pipeline not only enables stress quantification but also contributes to mental health technology by incorporating machine intelligence into mental state analysis.

### 2.1 Flow of experiment

The factory workers' stress assessment experiment is conducted in 9 episodes, details of which are discussed in the next section (section 2.2). In this experiment, crisp packets are delivered to the participant on a conveyor belt, each displaying a cognitive question. The cognitive questions are intended to increase mental workload while ensuring consistency among participants. A series of IQ-style problems, including geometric figure counting, visual pattern completion, symbolic numerical reasoning, and odd-one-out identification, are used to increase cognitive load. Such tasks require participants to use working memory, visual attention, reasoning, and decision-making processes while also performing packaging activities. The difficulty level is intentionally kept moderate to prevent extreme task failure while still inducing significant cognitive load. Each question has one correct answer, and participants are required to respond before progressing with the packaging task. A packaging workstation inspired by a factory is created using a Universal Robots UR5 collaborative robot and a conveyor belt system. Packets are delivered to participants one by one via the conveyor, and they pick up each packet and answer the cognitive question displayed on it before placing it in a box. Once the required number of packets (6) is collected, the box is placed in front of the cobot, which completes the packing process before the participant places it on the pallet. The UR5 cobot is programmed to operate according to the estimated completion time of a six-packet batch.

As illustrated in Figure 2A, this human-robot collaborative workflow simulates a realistic production scenario representative of industrial packaging and assembly environments. Figure 2B illustrates the experimental procedure, and Figure 3 shows the hardware architecture for conducting this experiment. Cognitive stress is evaluated by varying task complexity and speed, noise level, and light intensity.

**Figure 2:**
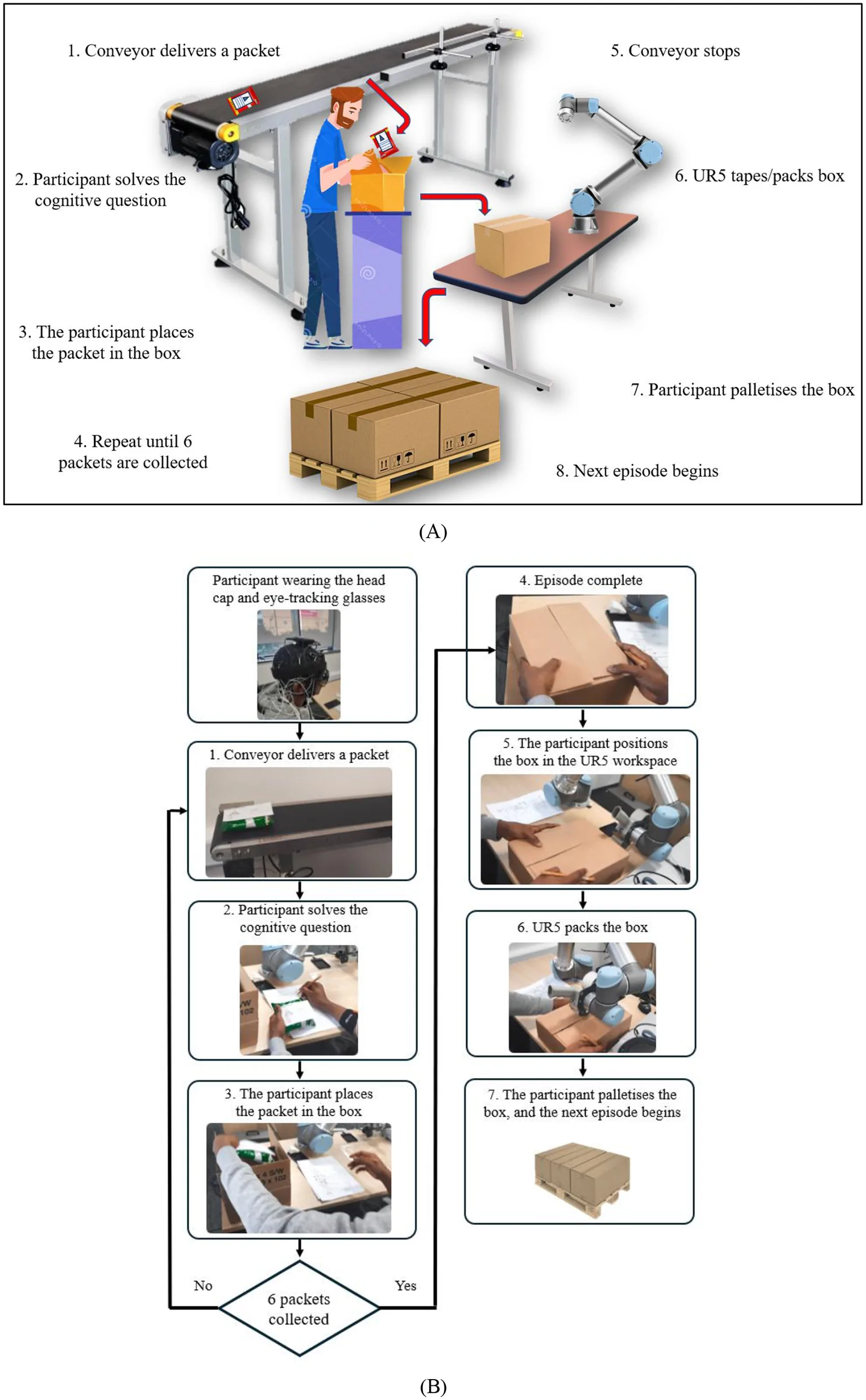
Factory workers' stress assessment experiment: **(A)** Workflow; **(B)** Experiment procedure.

**Figure 3:**
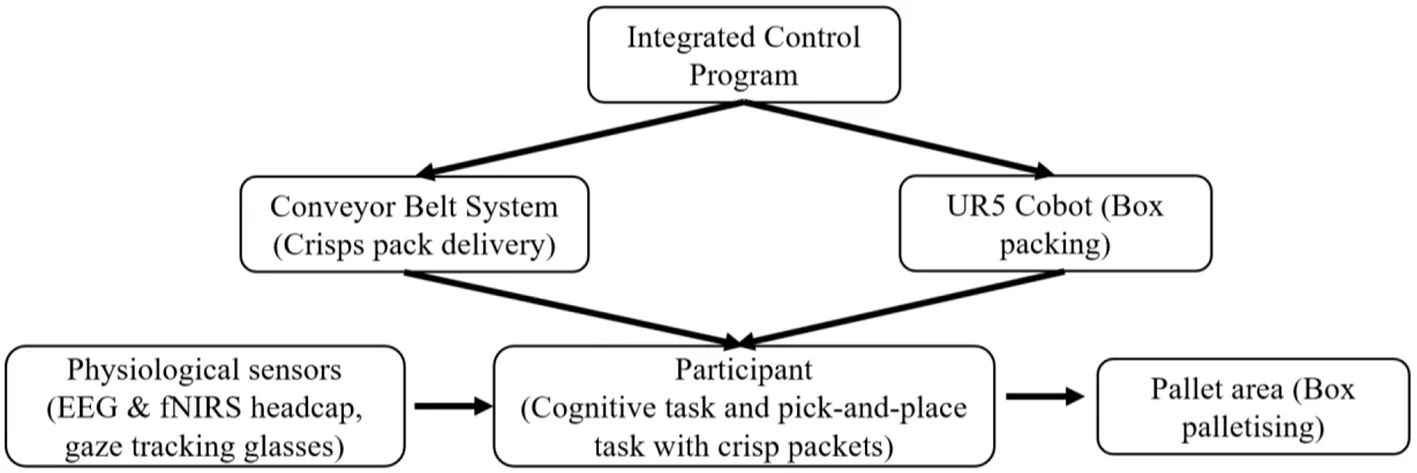
Hardware architecture for the factory workers' stress assessment experiment.

For the packet-counting mechanism, the conveyor is programmed to perform six delivery events in each episode, each corresponding to the delivery of one packet to the participant. Participants complete the cognitive task presented on each packet and place the packets in the box. The conveyor stops when the sixth delivery event is completed. The UR5 cobot is then activated to pack the box, while the participant remains idle until the task is complete. Following the robot operation, the conveyor is reactivated, and the next six-packet episode begins. This coordinated workflow has established a sequential human-robot interaction, with the participant performing cognitive, packaging, and palletizing activities and the UR5 performing the packing task. The synchronization of conveyor operation and robot movement ensured accurate packet counting, fixed batch sizes, and consistent human-robot interaction throughout the experiment.

A secondary task is introduced to increase task complexity. The episodes with high task complexity include the secondary task. As a secondary task, the participant receives a visual stimulus in the form of a damaged packet and must separate these from the intact ones. Damaged packets must not be placed in the box; doing so is counted as an error.

### 2.2 Experimental paradigm

The experiment is designed to record participants' brain activity continuously for 60 min. Before data collection, participants receive instructions, provide informed consent, and have all devices calibrated and fitted. The session begins with a 2-min rest period, followed by the first task episode of approximately 3.5 min. After each episode, participants complete the NASA-TLX and STAI questionnaires. The episodes are then conducted in the order shown in Figure 4.

**Figure 4:**
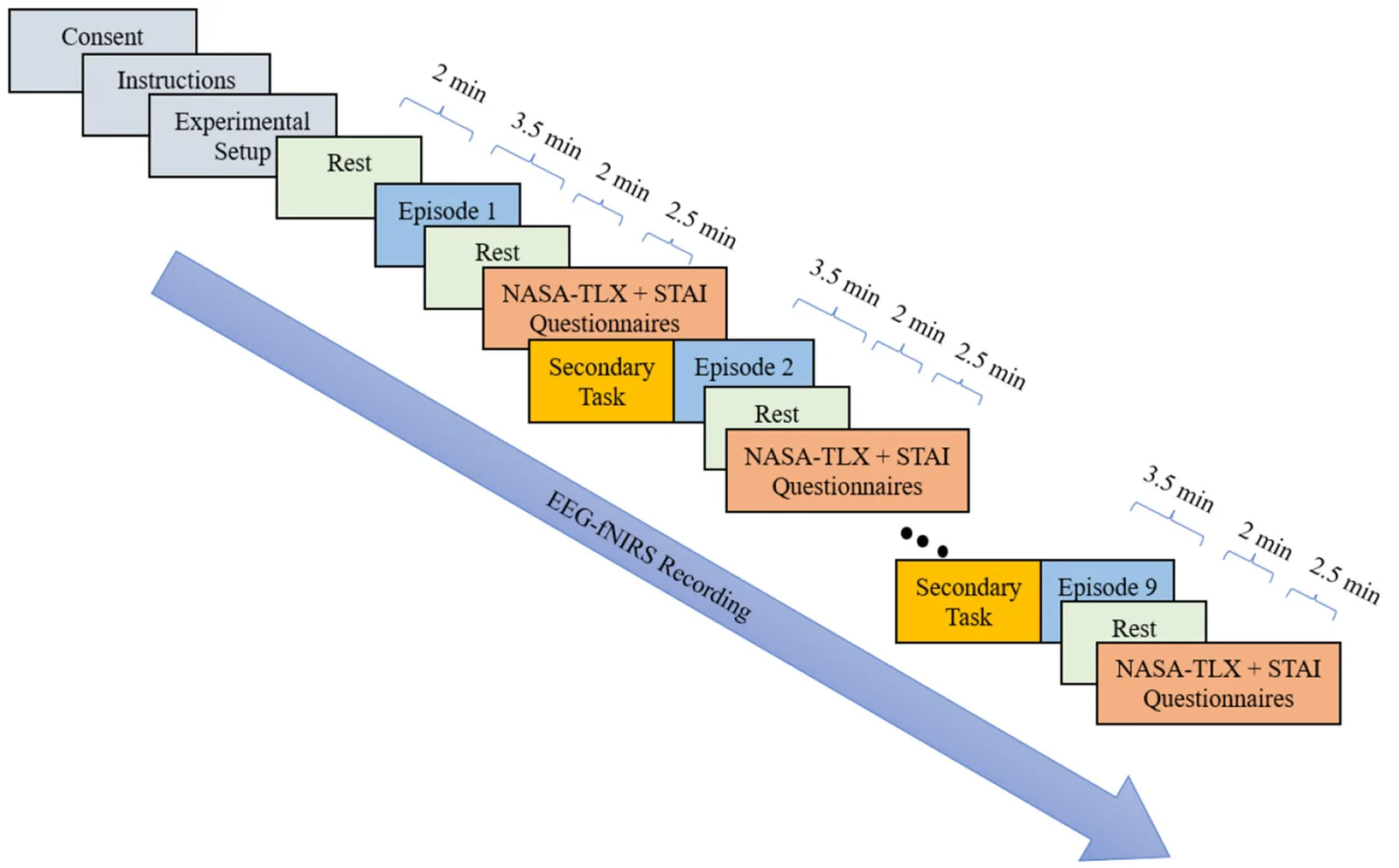
Experimental paradigm for the factory workers' stress assessment experiment. Nine task episodes (3.5 min each) with rest episodes (2 min each) and questionnaire-filling periods (2.5 min each) in between are demonstrated.

As shown in Figure 4 and detailed in Table 1, each episode is conducted under predefined low or high parameter settings. Task speed and task complexity are varied together to mimic realistic workload variations in industrial setups. This approach is based on findings from our previous study (Zakeri et al., 2023), where both variables illustrated similar effects on cognitive stress. Therefore, the present study focused on evaluating stress responses to the combined variation of task speed and complexity rather than investigating the individual effects, which have already been studied in our earlier study.

**Table 1:** Experimental episodes for the factory workers' stress assessment experiment, with “H” and “L” indicating high and low levels, respectively, for each process variable.

Episode no.	Task speed and complexity	Noise level	Light intensity
1	L	L	L
2	L	L	H
3	L	H	L
4	L	H	H
5	H	L	L
6	H	L	H
7	H	H	L
8	H	H	H
9 (Sub-episode: human-human interaction)	H	H	H

In the ninth episode, the cobot is replaced by a human operator. The condition is introduced to provide an exploratory baseline for understanding human-human and human-robot interaction. Light levels of 50–60 lux are considered low, and levels above 250 lux are considered high (HSE, 1987); noise below 55 dB is classified as low, while 80–85 dB is considered high (Regulations–HSE, 2025).

To keep the experiment consistent, the workflow has been kept the same for all participants; however, task memorization and habituation effects are reduced by varying experimental conditions (as can be seen in Table 1) and task content. There are nine episodes with varying process parameters, and each episode includes six cognitive questions with similar difficulty levels. Throughout the experiment, this strategy induced cognitive load while lowering the probability of task memorization and habituation.

### 2.3 Data acquisition

Twenty participants have taken part in this research. EEG, fNIRS, and eye-tracking data have been acquired during a human-robot collaboration task in a factory environment. EEG and fNIRS data are gathered to measure neural and hemodynamic activity, respectively. Due to the complexity, duration of the multimodal experimental flow and repetitive task simulations, the study has been conducted as exploratory research involving 20 participants. The objective of this study is to evaluate the viability and predictive capability of the proposed multimodal framework rather than establish population-level generalisability.

EEG signals are recorded using the TMSi Saga wireless system at a 4,000 Hz sampling rate (Jin et al., 2022), with 19 electrodes positioned according to the international 10–20 system. fNIRS data is acquired using the Artinis Brite device with 21 channels at a 75 Hz sampling frequency (Argyle et al., 2021), with transmitter-receiver pairs spaced 20–30 mm apart. Gaze tracking is conducted throughout the experiment using Tobii Pro Glasses 3, recorded at a sampling frequency of 50 Hz and 25 fps with a 1920 × 1080 resolution (Onkhar et al., 2024). Subjective measures are recorded after each episode using the NASA-TLX and STAI questionnaires.

### 2.4 Data pre-processing

A total of 20 participants (13 male and 5 female) were recruited using inclusion and exclusion criteria, with eligible individuals ranging in age from 18 to 55 years, had no color-blindness, and could complete the experimental activities. Individuals with a history of neurological diseases or the presence of cardiac implants were excluded from participating because these factors could alter the physiological measurements obtained throughout the experiment.

Multimodal data has been acquired from 20 subjects, yielding 175 samples. Each participant was required to complete 9 episodes, resulting in an expected total of 180 samples. However, one participant opted to withdraw from the experiment after completing 4 episodes. We retained data from the completed episodes, resulting in a total of 175 initial samples. A machine-learning sample represents a single experimental observation obtained from an individual participant during one episode. For each sample, EEG and fNIRS features are utilized, labeled using target variables (NASA-TLX and STAI scores), and subsequently employed for machine-learning model development. Following data quality assessment and the removal of samples affected by artifacts, missing values, or incomplete recordings, 147 valid samples were retained for machine learning analysis. For the classification models, stress levels were determined using STAI scores and grouped into three categories: low stress (82 samples, 55.8%), medium stress (31 samples, 21.1%), and high stress (34 samples, 23.1%). The final dataset was divided into training (70%, *n* = 103) and testing (30%, *n* = 44) subsets for model development and evaluation.

Both EEG and fNIRS data are pre-processed to obtain clean signals (Supplementary Figures 1, 2). EEG recordings are first cleaned using an ICA-based method to remove artifacts and non-brain activity, then down-sampled to 250 Hz and filtered with a 0.5–40 Hz band-pass filter to eliminate drift and high-frequency noise. The EEG frequency bands considered include delta (0.5–4 Hz), theta (4–8 Hz), alpha (8–12 Hz), beta (12–28 Hz), and gamma (28–50 Hz) (Zheng and Lu, 2015).

To obtain artifact-free fNIRS signals, the intensity is first transformed to optical density (OD). Motion artifacts are then rectified using a wavelet-based motion artifact correction approach, after which each channel is filtered between 0.15 and 0.5 Hz. Finally, the Modified Beer-Lambert law is applied to convert OD to HbO and HbR concentrations (Bonilauri et al., 2021).

### 2.5 Feature extraction

Features are extracted from the pre-processed EEG and fNIRS signals. For EEG, spectral features are computed by normalizing each clean signal and applying five Kaiser-window-based FIR filters to generate the standard EEG sub-bands (Supplementary Figure 3) (GitHub–Farhadabedinzadeh, 2025). The average band powers for five frequency bands are then calculated and used as spectral features.

Several statistical features, including mean, standard deviation, variance, kurtosis, skewness, root mean square, shape factor, impulse factor, first and second differences and their normalized forms, are extracted from the EEG data (Hasan and Kim, 2019; Shon et al., 2018). For fNIRS signals, both linear features (mean, skewness, kurtosis, standard deviation, variance) and non-linear features (entropy, fractal dimension (box-count), and detrended fluctuation analysis (alpha)) are computed (Shirvan et al., 2018).

### 2.6 Data selection

For EEG data, all recordings are initially imported into the Automagic toolbox (Pedroni et al., 2019), where signal quality is assessed using four measures: the ratio of bad channels, overall high-amplitude data (OHA), timepoints of high variance (THV), and channels of high variance (CHV). Using amplitude and standard-deviation thresholds of 30 μV, found suitable for this dataset, the toolbox classifies each recording as *Good, OK*, or *Bad*. This evaluation enables consistent comparison across recordings and supports logical inclusion or exclusion of data in subsequent analysis.

The pertinent EEG data is then selected by visually examining the clean data. The following criteria are applied:

- Only data labeled “Good” or “OK” by the Automagic toolbox are retained, as illustrated in Supplementary Figure 4.
- Only those participants' data are eliminated where all episodes are marked as “Bad”.
- Noisy channels identified through visual inspection are removed.
- Only the bad channels' feature data is excluded instead of discarding an entire episode.

For fNIRS data, the following criteria are adopted to select the relevant data:

- Bad channels affecting signal quality are identified through visual inspection.
- Only the features from bad channels are removed, rather than discarding entire episodes.
- Episodes with brief signal-loss or connection dropouts are excluded.

A few measures have been taken to further improve the outcomes:

- Rejecting the first 5 s from all episodes, as the participant is not performing any cognitive task during this period, and only the conveyor belt is in motion to deliver the crisp packet.
- An undefined gap, generated in the feature matrix due to rejecting bad channels, is filled in using the averaging approach.
- Min-max normalization (Arora et al., 2021) is applied to scale each feature in the matrix between 0 and 1, using the formula:
  $$\begin{array}{l} Z_{norm}=\;\frac{Z-Z_{\min}}{Z_{\max}-Z_{\min}} \end{array}$$ (1)
  Machine learning approaches, especially those relying on distance or gradient calculations, can be more biased toward features with wider numerical ranges; however, normalization ensures that each feature contributes equally (Singh and Singh, 2022).
- The ensemble model approach, covered in more detail in section 2.10, is used to determine the optimal feature combination.

### 2.7 Data labeling

Mental stress is quantified by mapping the feature matrix to two target scores: NASA-TLX (0–100) (Ramadhana et al., 2021) and STAI (1–4) (Fauquet-Alekhine et al., 2016). These scores serve as target variables for supervised machine-learning models in both regression and classification approaches. For regression, the feature matrix is labeled with the NASA-TLX score and the STAI score is rescaled to 0–100 to ensure comparability. For classification, NASA-TLX scores are grouped into three stress levels (Table 2). STAI scores are first mapped from 1–4 to 20–80 and then divided into three anxiety levels, as shown in Table 3 (Shirvan et al., 2018).

**Table 2:** Levels of stress based on the NASA-TLX score.

NASA_TLX score	Level of stress
0–50	Low
50–80	Moderate
80–100	High

**Table 3:** Levels of anxiety based on the STAI score.

STAI score	Level of anxiety
20–37	Low
38–44	Moderate
45–80	High

### 2.8 Machine learning for mental stress prediction

Machine learning algorithms are used to predict cognitive stress and anxiety based on physiological and subjective indicators. Both regression and classification approaches are applied, using 70% of the data for training and 30% for testing, with 5-fold cross-validation to reduce overfitting. The feature set consists of 147 samples, of which 70% (103 samples) are used for training, and 30% (44 samples) are allocated to testing. The partitioning was performed at the sample level. Therefore, samples from the same participant could be present in both training and testing sets. As shown in Supplementary Figure 5, the workflow includes data collection, feature extraction, model selection, training, testing, prediction, and optimal model deployment. Seven regression models, tree, linear regression, including SVM, Gaussian process regression, ensemble, neural networks, and kernel models, are evaluated. For classification, eight techniques are tested, including linear regression, SVM, tree, ensemble, Naïve Bayes, neural networks, kernel, and K-nearest neighbors (KNN). Additionally, other variants of these primary regression and classification methods are examined.

A range of EEG (spectral and statistical) and fNIRS (linear and non-linear) feature combinations is used to predict both NASA-TLX and STAI scores as targets. All regression and classification models are tested across multiple predictor sets, using accuracy as the metric for classification and RMSE for regression. For each set, the model achieving the lowest RMSE or highest accuracy is selected, and the best feature combination is selected for each target. Feature selection and ensemble learning are further implemented for EEG and fNIRS features only. Gaze-tracking and behavioral measures are studied separately and are not included as inputs to the machine-learning models.

### 2.9 Feature selection

Feature selection is crucial for developing effective machine learning models, as it identifies the most informative features while removing redundant ones. This process enhances accuracy, minimizes overfitting, speeds up computation, and improves interpretability, especially important for dealing with high-dimensional datasets. In this study, ReliefF (Reyes et al., 2015) and the *F*-test (Galvão et al., 2008) are used for regression, while Kruskal-Wallis (McKight and Najab, 2010), ANOVA (Elssied et al., 2014), Chi^2^ (Jin et al., 2006), and ReliefF (Spolaôr et al., 2013), are applied for classification.

The dataset is initially divided into training (70%) and testing (30%) subsets, and feature selection is performed on the training data only. The selected features are then used for model training and testing, preventing feature selection leakage.

### 2.10 Ensemble learning approach

Ensemble learning is a machine learning approach in which multiple base or weak learners are trained and their outputs combined to improve performance (Mahajan et al., 2023), as illustrated in Supplementary Figure 6. By aggregating diverse models, ensemble methods typically outperform individual models in accuracy, robustness, and resistance to overfitting. Although each model has its own advantages and disadvantages, combining them produces more stable predictions and reduces bias and variance (Webb and Zheng, 2004). In this study, four ensemble variants, averaging, weighted averaging, majority voting, and weighted majority voting, are employed to identify the best-performing configuration.

These ensemble techniques, including averaging, weighted averaging, majority voting, and weighted majority voting, improve model performance by utilizing the strengths of several base learners. The detailed results of the mental stress quantification framework applied to the stress assessment experiment for the factory workers are discussed in Section 3.

Another dimension of this study's scope is the development of a digital solution in the form of an Android application, *Stress Sense*, to monitor workers' stress levels, enabling preventive measures and seamless communication between workers and supervisors. For this purpose, the mental stress score is predicted using the best-performing machine learning model. The predicted stress score is translated into a visual stress scale, which is integrated within the Android app to support mental stress interpretation.

### 2.11 Gaze data analysis

Gaze data recorded using Tobii Pro Glasses 3 is analyzed following the workflow shown in Supplementary Figure 7. Gaze recordings are initially imported into the analysis software, and custom events and task episodes are aligned with the gaze recordings. Assisted mapping was applied to align gaze points with the gaze recording, followed by manual review to verify mapping accuracy and ensure data quality. Areas of Interest (AOIs) are then defined for the cognitive questions on the recorded video to interpret meaningful visual attention patterns. Custom tag groups are created to organize gaze data according to the experimental episodes. Required eye-tracking metrics, including fixation duration, visit duration, number of visits, and pupil diameter, and their varying patterns corresponding to different episodes, are visualized and studied. Finally, the selected metrics and annotated data are exported.

## 3 Results and discussion

Previous studies have approached stress assessment as a classification problem. In contrast, the proposed framework predicts continuous stress scores, which allows for a more appropriate portrayal of stress severity. While the predicted scores are based on validated subjective assessments rather than an objective physiological ground-truth stress index, the framework offers a quantitative measure of perceived stress intensity that may be more useful than classification labels.

The proposed framework for quantifying and digitizing mental stress in this paper is being practically demonstrated by the factory workers‘ stress assessment experiment. Participants have performed mentally challenging tasks in addition to a simple pick-and-place activity using crisps in a smart factory setting, and multidimensional data, including physiological signals and subjective stress levels, have been gathered. The experiment also involves continuous monitoring of environmental variables such as noise levels and lighting to explore their effects on cognitive strain. The stress quantification framework is implemented in multiple phases, including data preparation, feature extraction, mapping subjective scores to physiological feature matrices, and digitization using various machine learning techniques to determine the optimal one for deployment. Feature matrices are subjected to classification and regression algorithms to investigate the effectiveness of these methods. The stress conditions are digitized by comparing physiological indicators to self-reported stress scores, leading to the development of a data-driven stress scale. This scale enables the digitization of mental stress and provides a strong foundation for individualized stress tracking. The goal is to identify critical stress-related indicators and predict mental stress in a digitized format using machine learning to help employers and employees become aware of employees' mental condition.

### 3.1 Regression

The regression approach was initially applied to two datasets, one using the complete feature matrix (EEG and fNIRS) and another with data rejection based on data selection criteria, to compare the results and observe the effects of data selection. Using NASA-TLX and STAI scores as target variables, this comparison has enabled evaluation of how selective data inclusion influences the accuracy and reliability of regression results.

Initially, regression is applied separately to EEG spectral features, EEG statistical features, and fNIRS linear and non-linear features, followed by combined EEG and fNIRS predictors for both targets. All seven regression techniques are tested for each feature set, and the best-performing model is reported. Using the entire dataset without data rejection, the ensemble model achieves the lowest validation RMSE (18.48) and a test RMSE of 13.59 for the STAI score as the target, as shown in Supplementary Figure 8.

Supplementary Figure 8 indicates that fNIRS features alone achieve the best performance for the full, unrejected dataset, with the ensemble model performing optimally and highlighting the significance of linear and non-linear fNIRS features. Combining fNIRS with EEG statistical features shows the best performance for the STAI score as the target, emphasizing the importance of EEG statistical predictors. In contrast, EEG spectral features consistently produce higher RMSE values for NASA-TLX, while fNIRS features provide low RMSE for the STAI score as the target.

After applying data-selection criteria, multiple models are tested using EEG-only, fNIRS-only, and combined feature sets for both targets, NASA-TLX and STAI scores. The best performance is achieved by combining EEG statistical and fNIRS features, with the ensemble model (Figure 5) reaching a validation RMSE of 16.22 and a test RMSE of 16.18 for the STAI score as the target. Overall, EEG spectral features continue to show higher RMSE values than statistical features across both targets.

**Figure 5:**
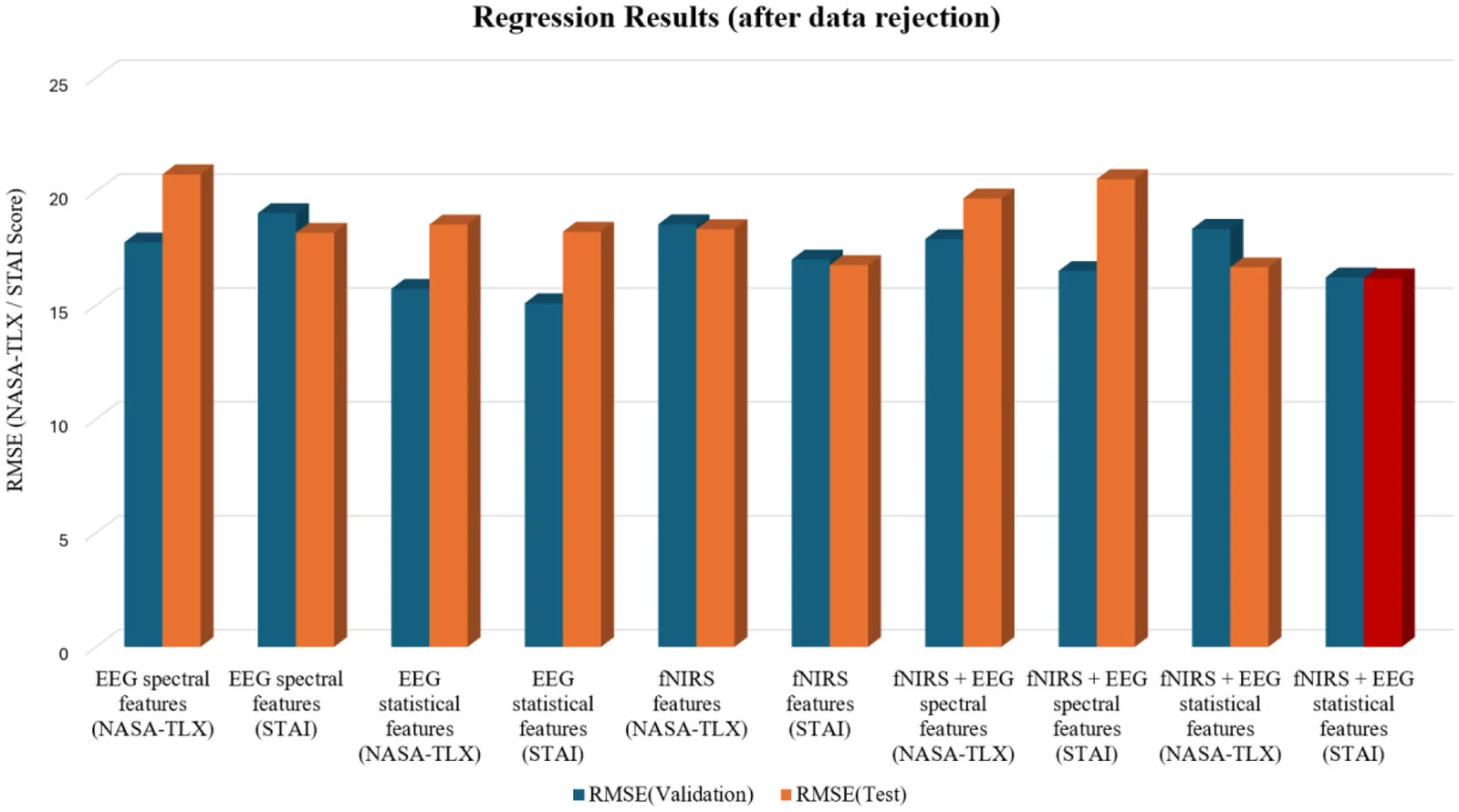
Regression results for the dataset after data rejection. NASA-TLX and STAI Scores are used as labels. The red bar illustrates the best-performing predictor set.

In the unrejected dataset, most feature sets produce a minimum test RMSE of around 18, with the best case reaching approximately 14. However, this does not indicate that the no-rejection technique is superior. Noise and artifacts can deceptively boost performance and reduce real-world generalizability. Data selection ensures cleaner inputs, lowers bias from poor-quality segments, and yields more consistent performance across feature sets (Urigüen and Garcia-Zapirain, 2015). Although the test RMSE is slightly higher after data selection, the reduced gap between validation and test suggests improved robustness and reduced overfitting (Montesinos López et al., 2022). Overall, data selection is essential for obtaining reliable and generalizable regression results.

A few strategies have been investigated to observe their effect on the outcomes, as mentioned in Section 2.6. After rejecting the initial 5 s of each episode in the fNIRS and EEG data, the feature matrix's undefined values are filled using the averaging approach. The best results were obtained by combining EEG spectral features and fNIRS features, with the STAI score as the target, and adopting Gaussian Process Regression as the regression algorithm (validation RMSE: 16.074), resulting in a minimum test RMSE of 11.747.

Normalization, as presented in Equation 1, is then applied to improve model performance by reducing scale-related bias in the selected feature set (combined fNIRS and EEG spectral features). Using the STAI score as the target, seven regression models were tested on the normalized data, with SVM achieving the best results (validation RMSE: 15.884; test RMSE: 11.563). The lower test RMSE indicates better generalization, showing that normalization can help maintain a consistent scale for all features while reducing bias from overly large-valued features. Overall, normalization enhances feature comparability, stabilizes training, and helps the model more effectively capture stress patterns in multimodal physiological data.

### 3.2 Classification

Similarly, the classification approach has been employed for two datasets: the dataset without any data rejected and the dataset selected based on data selection criteria stated in Section 2.6, to compare the outcomes. The results for the full dataset have been obtained first, followed by the selected dataset after data rejection. Like regression, the two targets for this strategy are NASA-TLX and STAI scores.

This investigation followed a similar approach to regression analysis. For the first dataset, EEG features are employed first, followed by fNIRS features, and then combined feature sets. As shown in Supplementary Figure 9, eight classifiers are tested for each predictor set, and the best-performing model is demonstrated. The highest test accuracy of 67.30% (validation accuracy 60.97%) is achieved using combined fNIRS and EEG spectral features with the STAI score as the target, and neural networks as the best-performing model.

Supplementary Figure 9 depicts the optimal performance achieved by combining EEG and fNIRS features for several classifiers, using the first dataset with the STAI score as the target, demonstrating the contribution of EEG features. In most cases, accuracy is higher using NASA-TLX as the target.

After applying data-selection criteria, the classification approach is repeated across all EEG, fNIRS, and combined feature sets using eight classifiers. As shown in Figure 6, the highest test accuracy of 65.9% (validation accuracy 63.1%) is achieved by combining fNIRS and EEG statistical features with the NASA-TLX target using neural networks, highlighting the significance of EEG statistical measures (Katmah et al., 2021) and both linear (Hong et al., 2018; Paulmurugan et al., 2021) and non-linear fNIRS features (Shirvan et al., 2018). Overall, most predictor sets show higher accuracy when NASA-TLX is used as the target.

**Figure 6:**
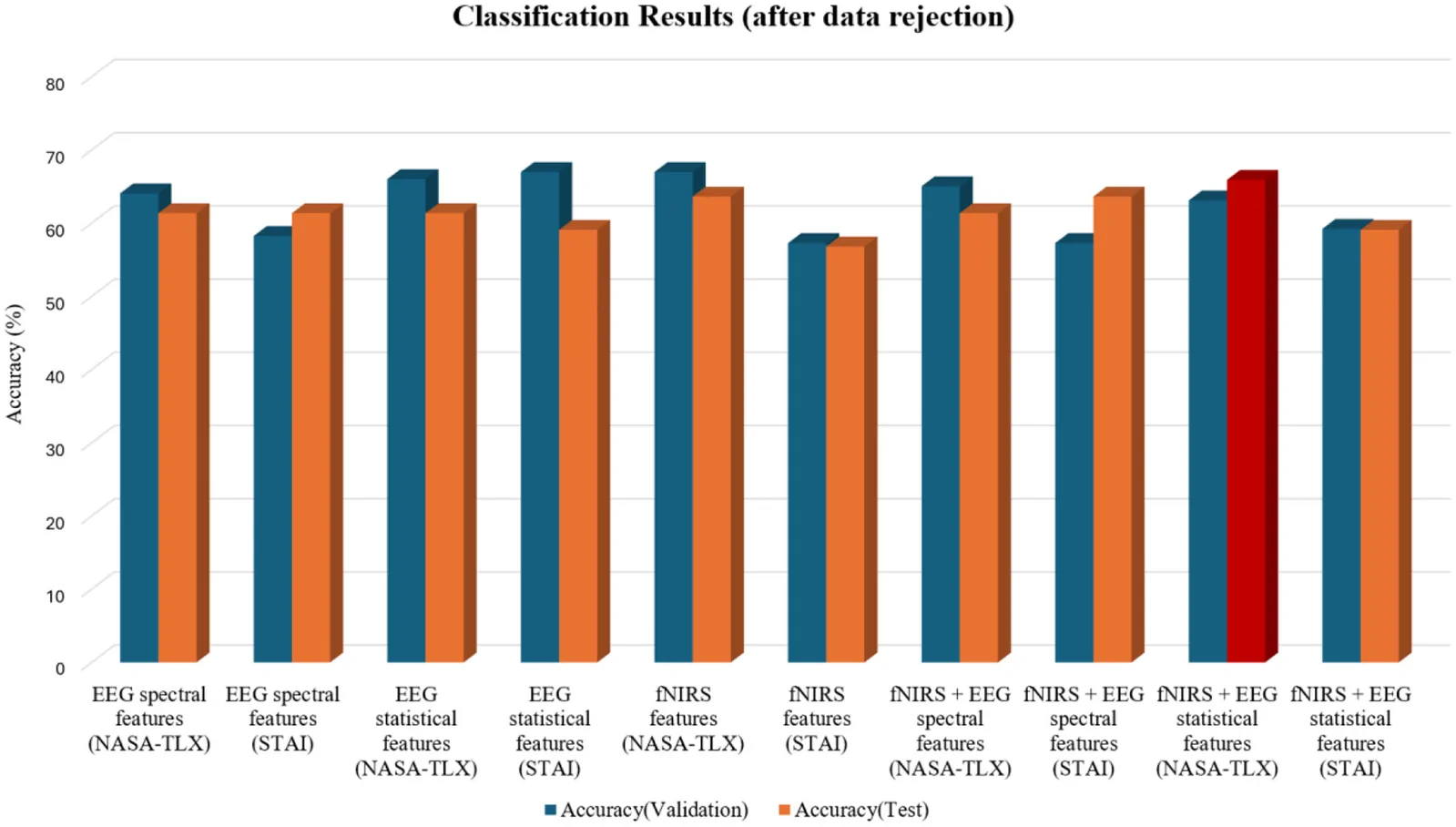
Classification results for the dataset after data rejection. NASA-TLX and STAI scores are used as labels. The red bar illustrates the best-performing predictor set.

After applying the data-selection criteria, validation accuracy decreased slightly compared to the unrejected dataset, but test accuracies became significantly more consistent. For example, the fNIRS + EEG spectral feature set (target: STAI score) shows a sharp drop from ~70% validation accuracy to ~55% on the test set, indicating overfitting. In contrast, the fNIRS + EEG statistical feature set (target: NASA-TLX score) achieves a consistent test accuracy of ~66%, closely matching its validation accuracy and demonstrating improved robustness. This pattern aligns with the principle that cleaner datasets may yield lower validation performance yet generalize better (Yang et al., 2022), as noise removal reduces overfitting and helps models learn meaningful patterns (Mwangi et al., 2014). Consequently, although accuracy decreases slightly after data rejection, the resulting models are more reliable and generalizable.

Further refinements, removing the initial 5 s of each EEG and fNIRS recording and filling missing values using averaging, improve performance. Using combined fNIRS and EEG statistical features with STAI as the target, SVM achieves a test accuracy of 79.55% (validation accuracy: 66.02%). After normalization, the same test accuracy is maintained (validation: 64.08%), suggesting enhanced generalization to unseen data. The significant difference between validation and test accuracy likely reflects that the validation set is not entirely representative of the underlying data distribution, or that the test set aligned better with the features used by the classifier.

### 3.3 Results after feature selection and ensemble approach

As outlined in Section 2.9, multiple feature selection algorithms are used to retain the most relevant features while rejecting redundant ones, for both regression and classification approaches. After applying the machine learning algorithms, an ensemble model is utilized to further reduce bias and improve robustness. The final model, chosen based on its optimal performance, is used to predict mental stress.

#### 3.3.1 Regression

For the regression approach, the combined fNIRS and EEG spectral feature set with STAI score as the target has shown the best performance. Two feature selection techniques, ReliefF and the *F*-test, are applied to identify the most significant features. Table 4 displays the number of features retained, the number of machine learning approaches used to apply the ensemble model and the resulting test RMSE values.

**Table 4:** Feature selection algorithms applied to the selected feature set (fNIRS and EEG spectral features).

Feature selection algorithm	Number of features	Number of classifiers for ensemble learning approach	RMSE (STAI score)
Without feature selection	284	3	10.9745
**ReliefF**	**213**	**3**	**10.856**
FTest	211	6	11.3269
After channel selection using ReliefF	210	5	11.1882

Bold values indicate the optimal result achieved among the evaluated feature-selection methods, corresponding to the lowest RMSE value.

Using ReliefF, with 0 as the threshold, has retained 213 of 284 features, producing the lowest test RMSE of 10.86 when combined with the ensemble model. The *F*-test, with the 10th-percentile threshold, has kept 211 features and achieved a higher RMSE of 11.32 when employed with the ensemble model. As ReliefF performed best, as shown in Table 4, it is used for channel selection. The channel selection criterion followed here is that if more than 4 features in a channel are rejected, the entire channel is removed. This led to the exclusion of one EEG channel (Fp2) and six fNIRS channels (Tx6–Rx4, Tx5–Rx5, Tx6–Rx5, Tx6–Rx6, Tx7–Tx6 & Tx8–Rx7), leaving 210 features and yielding a test RMSE of 11.19.

Without feature selection, the baseline RMSE is slightly below 11. Applying ReliefF reduces the RMSE to about 10.85, giving the best performance among all methods. In contrast, the *F*-test produces the highest RMSE at around 11.35, indicating weaker performance. After channel selection using ReliefF, the RMSE rises to approximately 11.2, still better than the *F*-test but not as strong as using ReliefF alone.

ReliefF outperforms the other feature-selection methods because it effectively handles complex, high-dimensional, and noisy multimodal data, capturing non-linear feature interactions that statistical tests like the *F*-test cannot (Pupo et al., 2013). Its robustness to noise and redundancy improves generalizability and reduces RMSE, achieving the best value of about 10.85. The strongest regression performance is obtained by applying ReliefF followed by an ensemble model using the combined fNIRS and EEG statistical feature set, which is then used to quantify factory workers' mental stress.

The proposed framework assesses perceived stress levels based on established subjective assessment scores and makes no claim to develop a universal physiological ground-truth measure of stress. As a result, the term “stress quantification” refers to the continuous estimation of stress scores rather than the direct measurement of an objective stress component. A continuous stress-level estimation framework has been developed, supporting H2, to record slow variations in worker stress that may not be visible through discrete stress classification techniques.

#### 3.3.2 Classification

Similar to the regression analysis, the combined fNIRS and EEG statistical feature set, with the STAI score as the target, is used for feature selection and ensemble modeling. STAI scores are used to determine stress levels in the classification model, which are categorized into three classes: low stress (82 samples), medium stress (31 samples), and high stress (34 samples) based on the thresholds shown in Table 3. The final dataset is split into training (70%, *n* = 103) and testing (30%, *n* = 44) sets for model development and testing. Four feature selection techniques (Kruskal-Wallis, ANOVA, Chi^2^, and ReliefF) are applied to select the most informative features. Table 5 compares the results after applying the feature selection algorithms followed by an ensemble model.

**Table 5:** Feature selection algorithms applied to the selected feature set (fNIRS and EEG statistical features).

Feature selection algorithm	Number of features	Number of classifiers for ensemble learning approach	Accuracy (%)
Without feature selection	417	3	81.81
Kruskal Wallis	316	7	79.54
ReliefF	197	5	75
ANOVA (10% of the highest rank value as threshold for exclusion)	274	5	79.54
ANOVA (10th percentile for exclusion)	267	7	79.54
**Chi**^**2**^ **(10% of the highest rank value as threshold for exclusion)**	**338**	**7**	**84.1**
Chi^2^ (10th percentile for exclusion)	331	7	79.54
After channel selection using Chi^2^ (10% of the maximum value as a threshold for exclusion)	351	7	77.27

Bold values indicate the optimal result achieved among the evaluated feature-selection methods, corresponding to the highest accuracy value.

For Kruskal-Wallis, features scoring below 10% of the highest rank are eliminated, leaving 316 of 417 features. ReliefF has used a threshold of 0, while ANOVA and Chi^2^ are tested with two thresholds: 10% of the highest rank and the 10th percentile. As shown in Table 5, the highest accuracy (84.1%) is achieved using Chi^2^ with the 10% rank threshold. The selected Chi^2^ features are then utilized for channel selection, applying the same rule as in the regression analysis; channels with more than four rejected features are removed. This resulted in retaining 351 of 417 features, with only one EEG channel (O_2_) and six fNIRS channels (Tx3–Rx2, Tx3–Rx3, Tx4–Rx4, Tx7–Rx6, Tx7–Rx7, and Tx9–Rx7) discarded. Using these selected channels, the final ensemble model has achieved a test accuracy of 77.27%.

Without feature selection, the ensemble classifier achieves a baseline accuracy of about 81%. Using Chi^2^ with a 10% rank threshold increases accuracy to approximately 85%, demonstrating its effectiveness in identifying relevant features and removing redundancy. ANOVA and Kruskal-Wallis yield similar accuracies of around 80%, while ReliefF performs the worst at roughly 75%. After channel selection based on the best Chi^2^ configuration, test accuracy decreases to about 78%, suggesting that some relevant features may have been eliminated.

Chi^2^ is particularly effective for categorical outcomes because it evaluates how strongly each feature's distribution correlates with discrete class separation (Peker and Kubat, 2021). In this experiment, it successfully identifies distinct EEG and fNIRS patterns across stress levels. Eliminating the lowest-ranked 10% of features reduces noise and minimizes overfitting, enabling the model to focus on the most informative predictors. This balance enhances overall classification accuracy by retaining relevant features while discarding redundant ones.

The overall findings for both approaches, regression and classification, emphasize that physiological features exhibited stronger predictive performance for STAI scores than for NASA-TLX scores. This implies that physiological features are more closely linked to subjective stress levels, as measured by the STAI score. Moreover, the weaker association observed for the NASA-TLX may reflect limitations arising from its multidimensional nature, as not all factors directly align with mental stress.

Based on the confusion matrix in Figure 7, class 1 (low stress) has performed well with an F1-score of 0.941, a recall of 0.960, and a precision of approximately 0.923. Class 2 (moderate stress) has demonstrated a moderate performance with an F1-score of 0.706, a precision of 0.750, and a recall of 0.667, highlighting some overlap with neighboring stress categories. Class 3 (high stress) achieved a precision of 0.700, a recall of 0.700, and an F1-score of 0.700, demonstrating balanced but comparatively lower performance than Class 1.

**Figure 7:**
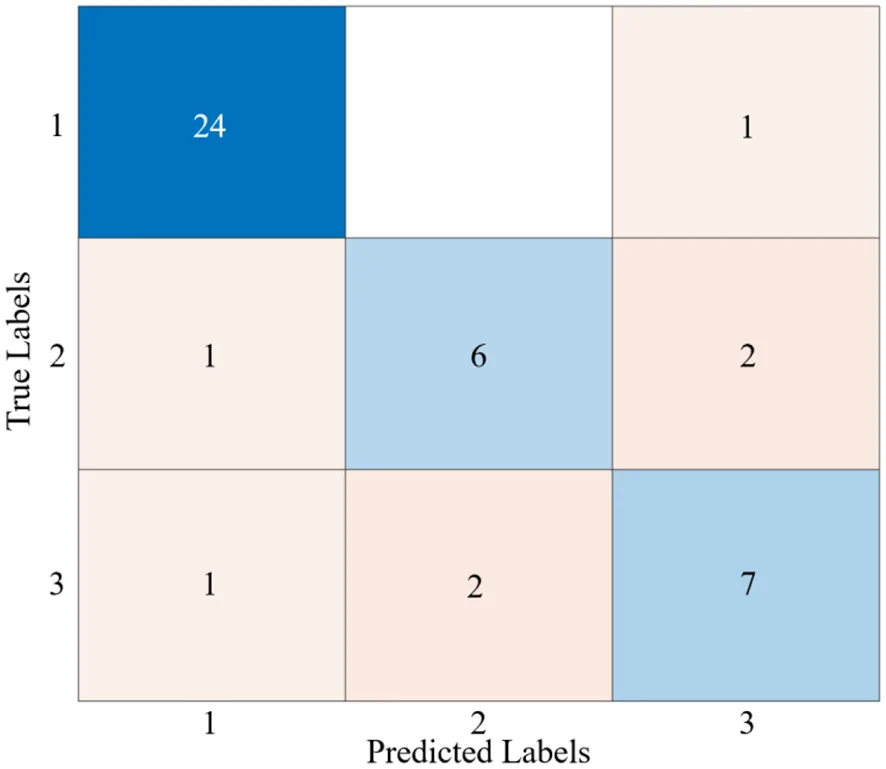
Confusion matrix for the best classification model for the selected feature set (fNIRS and EEG statistical features).

The classification outcomes indicate that the ensemble model integrated with Chi^2^ feature selection with a rejection threshold of 10% of the highest-ranked features has been particularly efficient in separating the low-stress class from the other stress categories. Specifically, 24 of the 25 low-stress samples have been correctly categorized, with only one sample incorrectly classified as high stress and none as moderate stress. For the moderate-stress class, 6 of the 9 samples have been correctly classified, while the remaining samples have been primarily misclassified as high stress, indicating a certain extent of feature overlap between the moderate- and high-stress levels. The high-stress class achieved the lowest classification performance, with 7 of the 10 samples correctly identified and the rest misclassified as either low or moderate stress. This behavior can be explained by resemblance in physiological responses between heightened stress levels and the relatively smaller number of high-stress samples existing in the dataset. Overall, the proposed Chi^2^ feature-selection followed by ensemble-learning framework effectively distinguished low-stress states and provided satisfactory differentiation between moderate- and high-stress levels. However, further feature set refinement may improve the discrimination of these adjacent stress classes.

### 3.4 Gaze tracking

Several eye-tracking parameters are explored in this study, including fixation duration, pupil diameter, visit duration, and number of visits. Results show that average durations of fixation, maximum duration of fixation, and average time of a visit are highest in the first episode and steadily decline by episode 9 (Figure 8), while the number of visits increases. Average pupil diameter is larger in low-light conditions (5–6 mm) and smaller in high-light conditions (4–5 mm). Each episode contains six cognitive questions, represented as Q1–Q6 in episode 1 (Figure 8A) and Q49–Q54 in episode 9 (Figure 8B).

**Figure 8:**
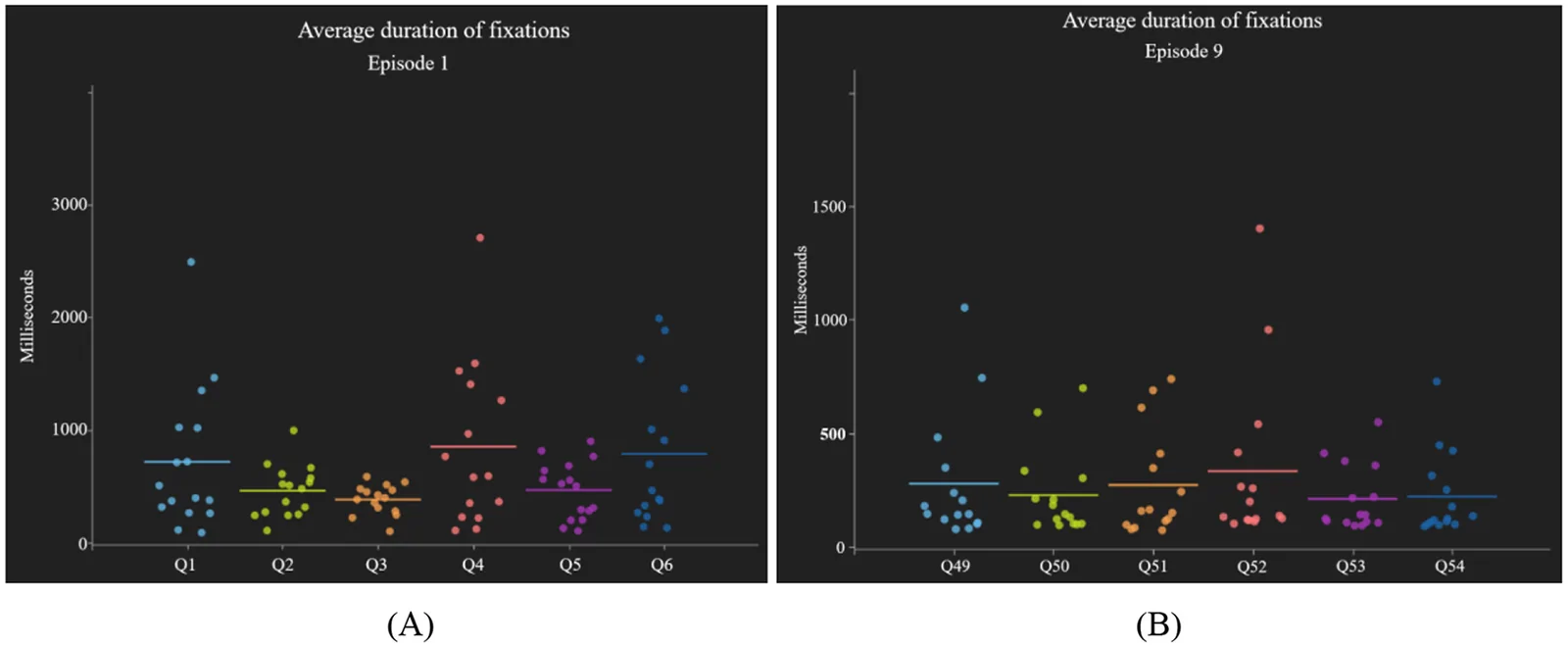
Average duration of fixation: **(A)** Episode 1, **(B)** Episode 9.

Fixation duration is higher for low and high STAI scores but lower for medium scores, and it generally increases with NASA-TLX across episodes (Figure 9). The number of fixations has decreased as both NASA-TLX and STAI scores have risen. Average pupil diameter has also declined with higher NASA-TLX and STAI scores. Additionally, fewer fixations have occurred for incorrect answers compared with correct ones.

**Figure 9:**
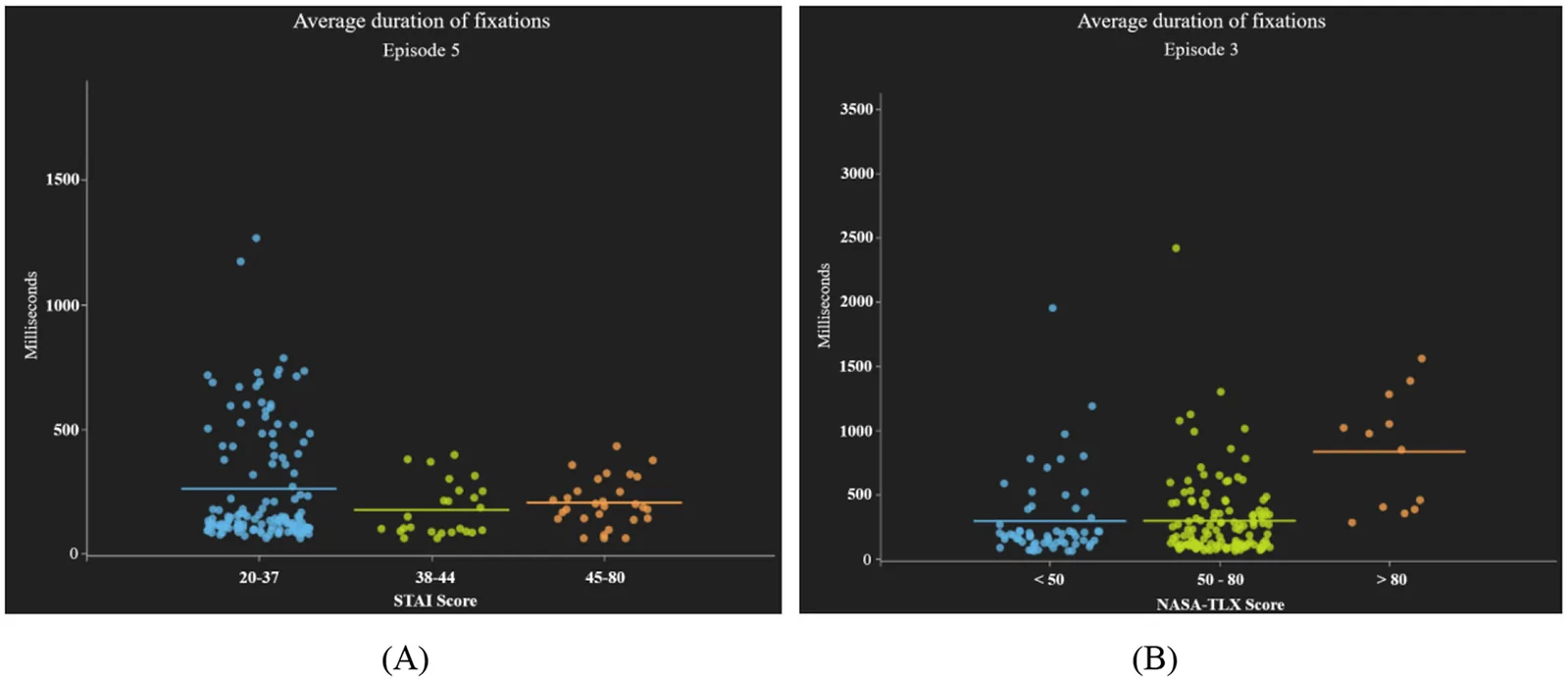
Average duration of fixation changing with **(A)** STAI score, **(B)** NASA-TLX score.

This research reveals clear patterns in visual attention and cognitive load. Longer fixation durations and visit times in the first episode indicate stronger initial cognitive effort, which diminishes as the task becomes more familiar, suggesting a learning effect. Pupil diameter variations between low- and high-light settings match expected physiological responses. The increased number of visits after the first episode indicates a shift in the visual search method. Associations between fixation metrics and STAI/NASA-TLX scores indicate that higher workload leads to longer but fewer fixations (Liu et al., 2022). Fewer fixations on incorrect answers than on correct ones suggest different information-processing strategies.

### 3.5 Subjective measures

#### 3.5.1 STAI score

STAI scores vary across episodes with changes in task and ambient variables, as shown in Figure 10. Noise and light show clear effects in the first four episodes. Episode 7 shows the highest score due to the most challenging conditions, i.e., high task speed and complexity, elevated noise, and low light, while episode 2 records the lowest score under favorable settings, supporting H1. Episodes 8 and 9 show comparable scores, indicating that replacing the robot with a human has minimal variance. The decreased stress trend in later episodes suggests a learning effect, as familiarity reduces mental tension.

**Figure 10:**
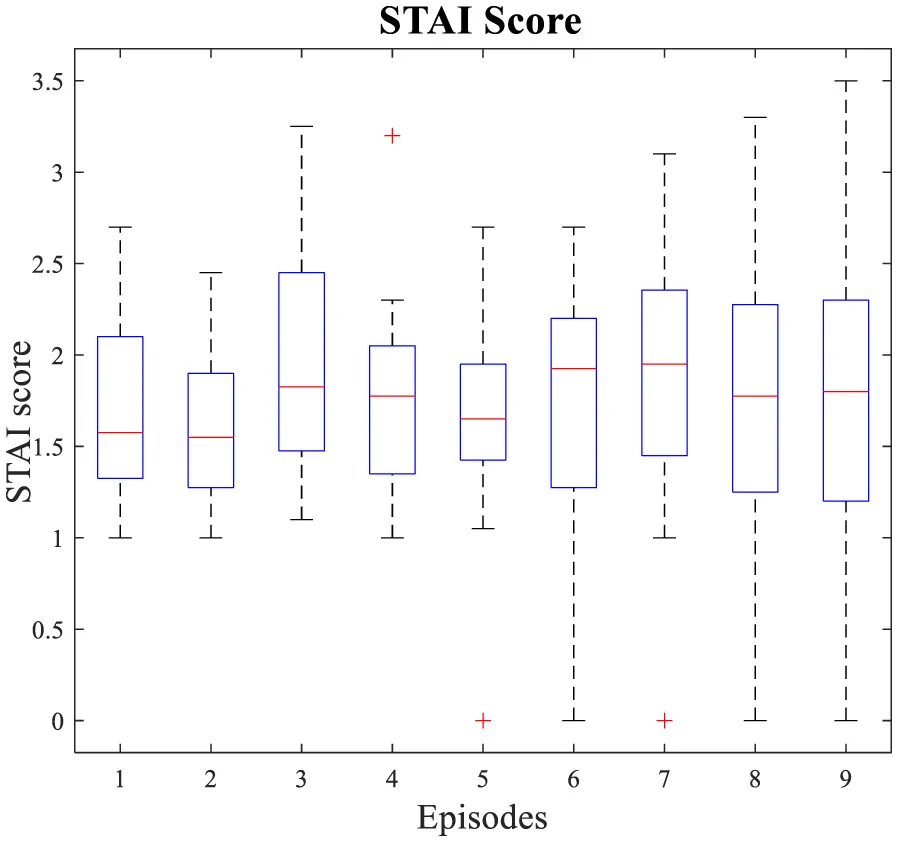
Box plot for STAI score demonstration across all task episodes. Variation in STAI score for all task episodes of the factory workers' stress assessment experiment is shown.

#### 3.5.2 NASA-TLX score

Figure 11 shows that the NASA-TLX scores for all episodes vary significantly depending on the task and the ambient conditions, supporting H1. The effects of noise and light are evident in the first four episodes, where workload perceptions fluctuate. Episode 9 shows the highest NASA-TLX score, indicating increased mental stress by the end of the experiment, while episode 1 records the lowest score, reflecting minimal cognitive load at the start. Overall, the NASA-TLX scores steadily increase across episodes, with early episodes, particularly episode 1, representing a low-strain baseline. Although noise and light influence stress in the initial episodes, the progressive increase highlights the combined impact of prolonged task engagement and growing fatigue on cognitive workload.

**Figure 11:**
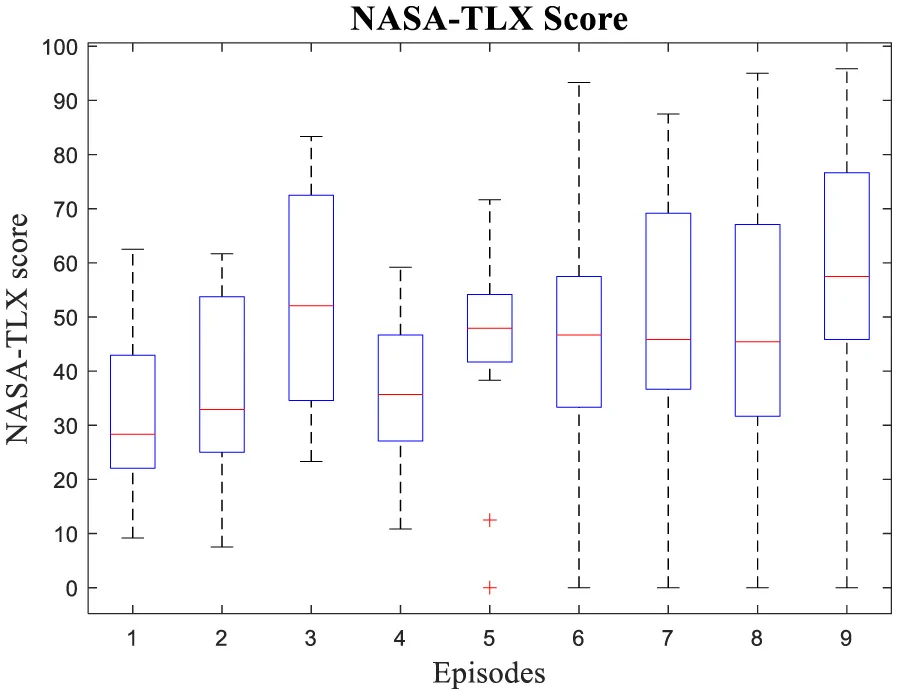
Box plot for NASA-TLX score illustration across all task episodes. The difference in the NASA-TLX score for all task episodes of the factory workers' stress assessment experiment is shown.

The results support all three research hypotheses. H1 is supported by the observed differences in behavioral and subjective stress measures across experimental episodes. H2 is endorsed by machine-learning results, which showed that the ensemble-learning approach performed best for both continuous stress-level estimation and stress classification.

## 4 Limitations and future work

Although this study shows strong potential for cognitive stress evaluation using neuroimaging, physiological data, and machine learning, several limitations must be acknowledged. The proposed framework does not operate in real time, limiting its use in dynamic, stressful environments, and currently depends on offline analysis followed by stress score prediction. The experiment has been conducted in a controlled simulation to study baseline stress patterns; it may not fully capture real workplace variability, such as unpredictability, environmental noise, or individual differences. The human-human comparison condition has been included only once as a single exploratory reference scenario rather than a fully repeated experimental condition. Therefore, the study does not provide a comprehensive comparison between human-human and human-robot collaboration modes. The dataset used is relatively small, increasing the risk of overfitting (Ghasemzadeh et al., 2024). The fact that a sample-level train-test split has been used for model validation instead of a participant-wise validation technique is another limitation of the current work. The training and testing datasets may have shared some participant-specific information. As a result, the reported results can be interpreted as subject-dependent model performance. The feature matrix has been normalized before machine-learning analysis to increase feature comparability and model stability. It should be highlighted that normalization has been conducted prior to the train-test split, meaning that the normalization parameters have been calculated using the complete dataset. While this has supported model development, it may have resulted in some data leakage between the training and testing datasets, which should be regarded as a limitation of this current study. The framework has only been tested with factory workers, restricting its robustness and generalizability across other occupational settings.

Future research can contribute to strengthen the stress-assessment framework by enabling real-time monitoring through an enhanced pipeline that integrates multimodal data with adaptive algorithms. Extending the framework to different occupational sectors would help evaluate its flexibility and improve its generalizability. Larger and more diverse datasets are also needed to extend the findings across various demographics, vocations and stress conditions. Improving physiological data quality remains important, as some artifacts could not be fully removed in this study; future studies should consider more accurate sensors with enhanced connectivity with the participant's body and experimental designs that minimize movement-related noise and artifacts. Future work can incorporate confidence interval estimation and statistical significance testing to further improve the robustness and reliability of the proposed framework. Future studies can also utilize subject-independent or leave-one-subject-out validation schemes to further evaluate the generalizability of the proposed framework. Future work can assess all experimental conditions under both collaboration paradigms, including human-human and human-robot interactions, to enable a more thorough evaluation of their respective effects on worker stress, performance, and wellbeing. Overall, the framework can be advanced by using higher-quality and larger datasets, and adaptive real-time methods to broaden its application across a wider range of occupational contexts.

## 5 Conclusions

This study presents and evaluates a novel continuous stress-level estimation framework for factory workers, employing machine learning to estimate cognitive stress levels from multimodal physiological measurements. The experimental setup combines IQ-style cognitive questions with a pick-and-place activity in an HRC environment. The results demonstrate the effectiveness of integrating EEG and fNIRS features with feature selection and ensemble learning. Chi^2^ has produced the highest classification accuracy, while ReliefF achieved the best regression performance, underscoring the importance of effective feature selection for robust modeling. Ensemble techniques further improved predictive accuracy, yielding the best results for both regression (RMSE: 10.86) and classification (accuracy: 84.1%), with the STAI score outperforming NASA-TLX as the target measure. An Android app-based digital solution has been developed to provide a visual tool to interpret mental stress in the form of a stress scale. Gaze-tracking analysis showed that fixation duration, number of fixations, visit duration, and pupil diameter varied with changes in process parameters. The average duration of fixation, number of fixations, and average pupil diameter are all affected by changes in subjective measures as well as the error rate. Changes in the levels of process parameters have an impact on NASA-TLX and STAI scores. Subjective variables have shown elevated mental stress under adverse conditions. Overall, human-machine interaction, combined with ambient variables such as low light (50 to 60 lux) and high noise (above 80 dB), raises the mental stress of a human participant.

This study advances occupational stress research by demonstrating that a multimodal stress-monitoring framework, combined with machine learning, can deliver meaningful insights and implementable solutions. The framework provides a solid foundation for future work, including real-time stress evaluation, long-term stress tracking, and broader application across diverse work environments. Overall, the novel framework presented in this study is an important step toward safer, healthier, and more sustainable workplaces, enabling proactive stress detection, intelligent decision-making, and timely interventions.

## Data Availability

The raw data supporting the conclusions of this article will be made available by the authors, without undue reservation.
