# Supplementary material for: Transforming neurophysiological data into measurable stress metric using machine learning-based stress quantification framework for smart factory workers
Source: Front Neuroergon. 2026 Oct 21;7:1871817. doi: 10.3389/fnrgo.2026.1871817 (PMC13732462; doi:10.3389/fnrgo.2026.1871817)
Supplement: Supplementary file 1 [file Data_Sheet_1.docx]

**Supplementary Figures**

**Supplementary Figure 1.** Flowchart of EEG data preprocessing. All the steps for EEG data preprocessing are shown.

**Supplementary Figure 2.** Flowchart of fNIRS data preprocessing. All the steps for fNIRS data preprocessing are shown.

**Supplementary Figure 3.** EEG spectral features extraction process. EEG spectral features are extracted using the steps demonstrated.

**Supplementary Figure 4.** Automagic toolbox for data quality assessment of EEG signals. Quality rating for EEG signals is assessed using the Automagic toolbox (Pedroni et al. 2019).

**Supplementary Figure 5.** Flowchart for Machine Learning. The Machine learning approach for predicting conventional measures employing physiological parameters is demonstrated.

**Supplementary Figure 6.** Flowchart demonstrating the Ensemble learning approach. The steps for employing the Ensemble learning approach are illustrated.

**Supplementary Figure 7.** Flowchart for gaze data analysis. All steps involved in gaze data analysis using Tobii Pro Lab are demonstrated.

**Supplementary Figure 8.** Regression results for the dataset without any data rejection. NASA-TLX and STAI scores are used as labels. The red bar illustrates the best-performing predictor set.

**Supplementary Figure 9.** Classification results for the dataset without any data rejection. NASA-TLX and STAI scores are used as labels. The red bar illustrates the best-performing predictor set.
